# Dynamic Vision Sensor-Driven Spiking Neural Networks for Low-Power Event-Based Tracking and Recognition

**DOI:** 10.3390/s25196048

**Published:** 2025-10-01

**Authors:** Boyi Feng, Rui Zhu, Yue Zhu, Yan Jin, Jiaqi Ju

**Affiliations:** 1 Shanghai Institute of Technology, Shanghai 201418, China; fby17359266910@gmail.com (B.F.); 18800359102@163.com (R.Z.); 2 Faculty of Art and Design, Yunnan University, Kunming 650091, China; june.zhu2025@outlook.com

**Keywords:** dynamic vision sensor (DVS), event-based vision, spiking neural networks, multi-scale attention, event convolution, real-time tracking, low-power inference, neuromorphic sensing

## Abstract

Spiking neural networks (SNNs) have emerged as a promising model for energy-efficient, event-driven processing of asynchronous event streams from Dynamic Vision Sensors (DVSs), a class of neuromorphic image sensors with microsecond-level latency and high dynamic range. Nevertheless, challenges persist in optimising training and effectively handling spatio-temporal complexity, which limits their potential for real-time applications on embedded sensing systems such as object tracking and recognition. Targeting this neuromorphic sensing pipeline, this paper proposes the Dynamic Tracking with Event Attention Spiking Network (DTEASN), a novel framework designed to address these challenges by employing a pure SNN architecture, bypassing conventional convolutional neural network (CNN) operations, and reducing GPU resource dependency, while tailoring the processing to DVS signal characteristics (asynchrony, sparsity, and polarity). The model incorporates two innovative, self-developed components: an event-driven multi-scale attention mechanism and a spatio-temporal event convolver, both of which significantly enhance spatio-temporal feature extraction from raw DVS events. An Event-Weighted Spiking Loss (EW-SLoss) is introduced to optimise the learning process by prioritising informative events and improving robustness to sensor noise. Additionally, a lightweight event tracking mechanism and a custom synaptic connection rule are proposed to further improve model efficiency for low-power, edge deployment. The efficacy of DTEASN is demonstrated through empirical results on event-based (DVS) object recognition and tracking benchmarks, where it outperforms conventional methods in accuracy, latency, event throughput (events/s) and spike rate (spikes/s), memory footprint, spike-efficiency (energy proxy), and overall computational efficiency under typical DVS settings. By virtue of its event-aligned, sparse computation, the framework is amenable to highly parallel neuromorphic hardware, supporting on- or near-sensor inference for embedded applications.

## 1. Introduction

In recent years, Spiking Neural Networks (SNNs) have attracted significant attention for providing energy-efficient, real-time solutions in event-driven perception tasks such as object tracking and recognition [1,2]. Beyond algorithmic accuracy, sensor-oriented systems emphasise low-latency inference, bounded memory footprint, predictable compute under varying event rates, and energy proportionality on neuromorphic and embedded platforms [3]. These properties make spike-based computation well suited to high-speed imaging under high dynamic range conditions in which frame-based pipelines often suffer from motion blur or saturation [4,5]. This perspective motivates architectures co-designed with the sensing pipeline and evaluated with sensor-relevant indicators (latency, event throughput, spike rate, and an energy proxy), positioning SNNs as a compelling alternative for edge-deployed tracking and recognition. The objective of this study is to identify the most effective methods for performing inference at the sensor or in close proximity to the sensor, while adhering to stringent energy and latency constraints. The utilisation of event sparsity, microsecond time stamps and polarity encoding ensures that computation is only performed when informative changes occur, thereby reducing redundant operations and power consumption.

Despite the advantages of Spiking Neural Networks, several challenges remain in optimising their training and handling the spatio-temporal complexity of event-driven data. A significant challenge pertains to the training of SNNs, which is hindered by the non-differentiability of the spike activation function [6,7]. Early research attempted to apply traditional backpropagation (BP) algorithms. However, significant challenges were encountered due to discontinuities in the activation function, which made gradient computation unfeasible [8]. To address this issue, surrogate gradient methods were introduced to approximate the gradients, thus enabling the application of BP to SNNs [9,10]. These methods provide continuous approximations to the spike function, thereby overcoming the gradient issue and facilitating more effective training. Training on asynchronous event streams is non-trivial, yet the use of surrogate gradients, modules that leverage sparsity and a loss that weights events makes optimisation tractable. The efficiency claim refers to inference, where sparse spikes reduce active operations and memory access.

**Figure 1:**
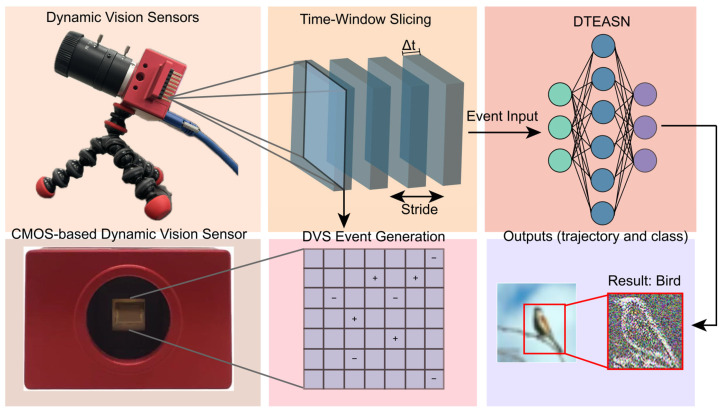
Overview of the DVS-driven sensing pipeline and the DTEASN framework. Dynamic Vision Sensors emit asynchronous polarity events with microsecond timing. Events are grouped in time windows and converted to a sparse spatiotemporal representation. The framework applies event-driven multi-scale attention and spatio-temporal event convolution, followed by a pure spiking backbone. Outputs comprise a category label and a target trajectory. The diagram highlights the alignment with sensor properties—asynchrony, sparsity, and polarity—to support low-latency, resource-efficient inference.

The potential of SNNs is particularly evident when paired with Dynamic Vision Sensors (DVS)—neuromorphic image sensors that capture asynchronous events corresponding to scene changes with microsecond-level latency—thus ensuring high temporal resolution and low latency [11,12,13,14]. In a typical representation, each event is denoted as e = (x, y, t, p), where (x, y) is the pixel location, t is the timestamp, and p is the polarity. An event is an atomic brightness change that occurs at one pixel and one time; recognition and tracking aggregate many events from many pixels over time, rather than following a single pixel. Figure 1 summarises a DVS-driven neuromorphic sensing pipeline and a pure spiking neural network framework (DTEASN), covering hardware, event generation, time-window slicing, spiking inference, and object-level outputs. However, integrating event cameras with SNNs is challenging due to the sparse and irregular nature of the event data, which makes it difficult for traditional SNN models to process these events efficiently in real time [15,16,17]. To address this challenge, recent research has investigated the application of rate coding and transformer-based attention mechanisms to effectively manage sparsity and enhance feature extraction from event streams [18,19,20]. While these approaches have improved accuracy by focusing on significant events and optimising the encoding process [21,22,23], the full potential of SNNs in real-time applications remains unrealized, particularly when sensor characteristics (asynchrony, sparsity, and polarity) are not explicitly taken into account.

Conventional frame-based pipelines based on reconstructed frames, optical flow trackers or recurrent models for dense video require substantial computation and memory traffic because sampling is synchronous and background pixels are redundant. The DVS-aligned spiking pipeline exploits sparse event streams and binary spike operations at inference, which lowers computational activity and improves the energy and latency profile on edge platforms.

Dynamic Vision Sensors find application in a variety of time-critical contexts, including high-speed robotics, HDR automotive perception under flicker and in low-light conditions, gesture-driven human–computer interaction, UAV navigation, and industrial inspection. The temporal resolution offered by these models is in the order of microseconds, the dynamic range is wide, and the outputs are sparse without motion blur. However, a gap remains in the model: inference must recover spatio-temporal structure efficiently on constrained edge hardware. It is therefore proposed that a sensor-aligned, pure spiking pipeline be adopted, with the objective of reducing frame and tensor overhead while preserving low latency and favourable energy behaviour. The effects of this approach on latency, event throughput, spike activity and an energy proxy are reported and discussed [12]. In this context, high dynamic range describes the imaging conditions rather than the scene. Sensor and data properties used in this study, including spatial resolution, polarity channels and temporal windowing, are summarised in Section 3.1, and full reproducibility details are provided in Appendix A.

Spiking neural networks are of increasing importance, both as standalone models and as the computational core for event camera perception. Recent neuromorphic platforms, including programmable digital chips such as TrueNorth, Loihi 2 and SpiNNaker 2 [24,25,26], as well as CMOS memristive arrays and superconducting Josephson junction devices, demonstrate practical routes to energy and area efficiency [27,28]. It is important to note that this study is software-only. The approach adopted is to implement a sensor-aligned, purely spiking recognition backbone with lightweight tracking, in order to meet the edge constraints of event-driven updates, sparse activity and limited memory. In addition to accuracy, the present study reports latency, event throughput, spike activity and an energy proxy to reflect these constraints. Both task accuracy and resource proxies aligned with event-sparse inference, including spike activity and an energy proxy, are reported in the Results section to substantiate the efficiency claim.

**Figure 2:**
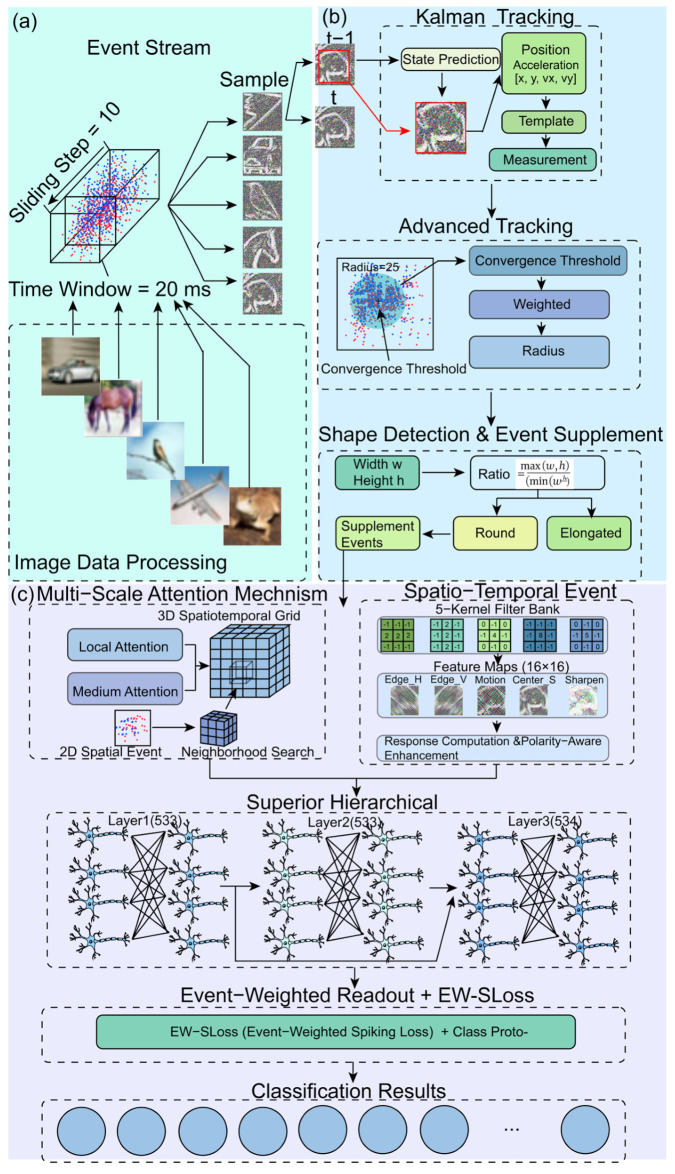
Overall framework for event-driven object tracking and learning.

Event-camera tracking in real time encounters abrupt target and ego motion that produce sparse yet bursty events, large and rapid scale or aspect changes, partial or full occlusions, and strong distractors in high-dynamic-range backgrounds; event rates vary over time, while memory, throughput, and energy on embedded platforms remain limited, so inference must remain stable at low latency. Let the DVS stream be asynchronous events $e_{i}=(x_{i},y_{i},t_{i},p_{i})$ with an initial target state $s_{0}=(c_{x},c_{y},w,h)$ at $t_{0}$. At time t, the tracker estimates $s_{t}$ from events in a sliding window [t−Δ,t] according to $s_{t}=T\left(\left\{e_{i}|t-\Delta \leq t_{i}\leq t\right\}\right).$ The objective is to minimise localisation error under a bounded latency budget. The estimate uses a constant-velocity prior for stability and measurements derived from the clustering and event-driven attention features described in Figure 2 and Section 3, matching the notation already introduced in the method.

To address the challenges of real-time object tracking, this paper introduces the Dynamic Tracking with Event Attention Spiking Network (DTEASN). As a pure SNN framework, DTEASN eliminates the computational overhead of traditional CNN operations, thereby reducing GPU dependency [29,30], and aligns the processing with DVS signal characteristics via time-window slicing and polarity-aware operations. DTEASN incorporates a multi-scale, event-driven attention mechanism designed to focus on the most salient portions of the event stream, thereby enhancing feature extraction. Additionally, a spatio-temporal event convolver is introduced to optimise processing by capturing both spatial and temporal features. Another significant innovation is the Event-Weighted Spiking Loss (EW-SLoss), a loss function that enhances tracking accuracy by differentiating between relevant and irrelevant events and improving robustness to sensor noise. The framework also includes a lightweight event tracking mechanism to reduce computational burden and a custom synaptic connection rule to optimise information flow. We evaluate DTEASN on event-based recognition and tracking benchmarks, and report sensor-relevant system indicators (latency, event throughput (events/s), spike rate (spikes/s), memory footprint, and a simple energy proxy), showing superior performance in terms of accuracy, energy consumption, and computational efficiency compared with conventional methods. Classification uses the category sets defined by the benchmark datasets throughout the study, and species level labels are not considered.

The primary contributions are summarised below:

(1) Innovative attention and convolution structures: Sensor-aware spatio-temporal multi-scale attention and event convolution that are aligned with DVS event statistics to enhance feature extraction from raw event streams.
(2) Event-Weighted Spiking Loss (EW-SLoss): A loss function that improves accuracy by prioritising informative events and enhancing robustness to sensor noise for event-driven SNN.
(3) Lightweight event tracking mechanism: An efficient tracking module that reduces resource usage while maintaining real-time performance for embedded sensing.
(4) Pure SNN architecture: A CNN-free spiking architecture that reduces GPU dependency and better matches asynchronous DVS inputs.

## 2. Related Work

### 2.1. Dynamic Vision Sensors and Event Representations

Dynamic Vision Sensors (DVSs) emit asynchronous polarity events $\;e=\left(x,y,t,p\right)\;$ at microsecond latency and high dynamic range, yielding sparse spatio-temporal streams that differ from frame images. In order to interface dense backbones, a significant number of studies have formed frame-like surrogates, including event count images, time surfaces/last-timestamp maps, and voxel grids [31] with temporal slices. However, these can dilute timing sparsity, raise latency, and increase compute on edge devices. The sensor-aligned time-window slicing technique has been demonstrated to preserve event ordering and polarity statistics, whilst also mitigating blurring effects caused by rapid movement. It is evident that a number of refinements are shared by the majority of approaches, including ON/OFF channels, local spatio-temporal neighbourhoods, and event-density normalization. In practice, sensor-aligned slicing is the preferred option when low latency and resource efficiency are required [15,20].

### 2.2. Attention Mechanisms in Spiking Neural Networks for Event-Based Vision

Recent studies have integrated attention mechanisms into Spiking Neural Networks with a view to enhancing the processing of dynamic and sparse event-driven data. These mechanisms enhance temporal and spatial feature extraction, which is critical for real-time applications. For instance, Xie [32] and Hu [33] demonstrated clear benefits for efficient event stream processing and feature selection. Temporal and spatiotemporal attention in DVS-based tasks facilitates superior management of sparse data with high temporal resolution [34]. The following evidence has been presented: an event-driven, multiscale attention system is designed, the weights assigned to each event being derived from temporal proximity to its neighbours, spatial coherence with the local motion pattern, and polarity consistency. The resulting weight is utilised in two distinct aspects of our framework: firstly, it facilitates the scaling of synaptic inputs within the spiking backbone, thereby ensuring that integration is focused on informative structures; secondly, it enables the reweighing of the training objective through the Event Weighted Spiking Loss approach, as outlined in Section 3. Liu [35] further demonstrated that allocating attention enhances the predictive accuracy of spatiotemporal forecasting, which is consistent with our objective of enhancing real-time performance [36,37].

### 2.3. Loss Functions for Spiking Neural Networks

The function of loss in determining the manner in which spiking networks allocate credit over time and space is a fundamental aspect of their operation. This process involves the modulation of membrane dynamics to facilitate the acquisition of useful structure from event streams. By specifying which discrepancies are to be penalised, the objective is to deliver surrogate gradients to the spike generation process, to align spike timing with targets, and to regulate firing so that informative activity is retained while noise is suppressed. The validity of these roles is supported by prior studies: residual formulations guide error propagation and accelerate convergence [38]; comparative analyses clarify how accuracy, calibration and sparsity trade off in practice [39]; sequence objectives improve long-horizon credit assignment and forecasting accuracy [40]; image-oriented penalties sharpen feature selectivity and reduce spurious activations [41]. In the present model, an event-weighted spiking loss is employed to match Dynamic Vision Sensor statistics. Each event is assigned a normalised weight that reflects temporal proximity, spatial coherence, and polarity consistency. The per-event error is multiplied by this weight and subsequently accumulated across the slice. The construction in Equations (13)–(15) implements this mechanism. The effect of this process is that coherent clusters contribute more strongly to parameter updates, isolated or contradictory events contribute less, and the same weights gate synaptic inputs so that inference focuses on the structures that drive learning. This, in turn, improves optimisation stability and predictive accuracy without additional computational cost.

### 2.4. SNN-Based Object Tracking and Recognition

Recent advancements in Spiking Neural Networks (SNNs) have demonstrated their efficacy in object tracking and recognition tasks. Yan [42] proposed a lightweight neural network (NN) for efficient object tracking. This has been enhanced by integrating spiking neural networks (SNNs) for real-time, low-power applications [43]. Pan [44] utilised a specific type of neural network, known as a spiking neural network (SNN), in conjunction with dynamic programming to facilitate vehicle tracking control. This study underscores the promise of SNNs in enabling real-time vehicle tracking applications. As demonstrated by Liu [45], the utilisation of SNNs has been shown to enhance the processing of motion for the purpose of visual recognition tasks. In their 2025 study, Aitsam [46] proposed a novel integration of dynamic attention mechanisms with event-based vision systems, with the objective of enhancing the capabilities of multi-object tracking. The extant literature emphasises the significance of SNNs in enhancing object tracking performance in dynamic environments.

## 3. Method

### 3.1. Sensor-Aligned Framework for DVS Event Streams

The input to the framework is constituted by a sequence of events produced by a Dynamic Vision Sensor. Each occurrence is delineated by a set of pixel coordinates, a timestamp with microsecond resolution, and a polarity indicator. The data transmission is segmented into predetermined temporal windows, with each window measuring 20 milliseconds and separated by a stride of 10 milliseconds. Within each designated time period, timestamps undergo a process of normalisation. Two polarity channels are maintained for ON and OFF events. A causal voxel grid is constructed over space and time to aggregate events while preserving temporal order. The occurrence of spurious isolated events is suppressed, and the total event count per window is constrained to ensure the exclusion of outliers. It is noteworthy that these settings are consistently maintained across all datasets utilised in the study, aligning with the examples depicted in Figure 2.

The processing of DVS event streams is conducted from start to finish, employing time-window slicing and polarity-aware operations to ensure the preservation of the asynchronous timing and sparsity characteristics inherent to DVS signals.

The overall framework depicted in Figure 2 integrates several novel modules for event-driven object tracking and learning. Initially, the event stream is processed by means of sampling the incoming events within a 20-millisecond time window, with a 10-millisecond sliding step. A validation sweep over window and step sizes is summarised in Table 1. These events are then mapped into a 3D spatiotemporal grid [47], which captures both spatial and temporal information, forming the foundation for accurate tracking and feature extraction. For each window [kΔt,kΔt+W) we process all events across the sensor that fall into the window. In other words, the slice is the set of events from all active pixels in that interval, which forms the basis for tracking and recognition.

At inference, the process entails the conversion of each event window into a voxel representation, followed by the application of the attention module and the spatio-temporal event convolver. The resulting features are temporally aggregated and forwarded to two heads. The classification head is responsible for producing class logits that correspond to the dataset level categories. The predicted label is the class that exhibits the highest logit. The tracking head generates the target trajectory, which is stabilised by the Kalman block as outlined in the framework. It is imperative to note that this procedure is applicable to all datasets utilised in the experimental process.

The Shape Detection and Event Supplement block is responsible for the connection of raw event clusters with the tracking and readout heads. The process of shape detection involves the aggregation of neighbouring ON and OFF events within each designated time window. This is followed by the estimation of a coarse contour and a centroid. The measurement is then spatially supported, thereby stabilising the Kalman update. The function of the event supplement is to interpolate short gaps when the inter-event interval in a slice becomes large or when occlusion disrupts clusters. Interpolation is constrained by a small spatial radius and a short confirmation window derived from the bounding-box diagonal and the constant-velocity prior. The additional points serve to preserve the asynchronous nature of the stream whilst maintaining polarity consistency. Their contribution is down-weighted by the attention module, thus ensuring that genuine measurements prevail. In practice, the block has been shown to reduce identity switches under rapid scale or aspect changes, and to lower the variance of the readout with negligible computational cost.

A key component of the framework is the Kalman Tracking Block, which predicts the object’s state, including position and velocity components $\left(x,y,v_{x},v_{y}\right)$ [48]. The predicted state is then refined using a measurement update, ensuring that the object’s trajectory is accurately estimated over time. Concurrently, the Advanced Tracking Cluster employs a radius-based clustering technique to group the events based on proximity, utilising a convergence threshold. This method enables efficient object identification by calculating the weighted centre and performing a radius search for neighbouring events, ensuring robust tracking in noisy environments.

The Event-Driven Multi-Scale Attention mechanism is a sophisticated computerised system that processes the event stream at multiple scales, thereby capturing critical spatial features at both fine and coarse levels. This mechanism enhances the feature extraction process by focusing on the most relevant regions of the spatiotemporal grid. Concurrently, the Spatio-Temporal Event Convolver employs convolution kernels on the event data to extract essential features, including edges, motion, and centres. The resulting feature maps undergo response computation and polarity-aware enhancement, ensuring that the most significant events are given higher priority in the tracking process.

The extracted features are then processed by the hierarchical spiking network, which refines the tracking results across multiple layers and captures both fine-grained details and broader object dynamics. A distinguishing element of the framework is the Event Weighted Spiking Loss, which assigns higher importance to events with greater spatiotemporal relevance. Here, spatiotemporal relevance denotes the evidential strength of an event measured by three factors: closeness in time to neighbouring events within the current slice, coherence in space with the local motion pattern estimated from the discrete velocity and acceleration described in Equations (4) and (5), and consistency of polarity with the dominant local contrast. These factors are combined and normalised to produce an attention weight $a_{i}$ as formalised in Equations (9)–(12), and this weight both scales the synaptic input during inference and multiplies the per-event error in the loss defined in Equation (15). The Event Weighted Readout then aggregates the weighted responses with class prototypes to obtain the final classification, yielding a lightweight design that supports real-time event tracking with modest computational resources. Instrument note. Event data were acquired with a DAVIS346 event+frame camera (iniVation AG, Zurich, Switzerland); experiments used the vendor DV-Platform where applicable.

### 3.2. Event-Driven Object Tracking

The tracking method processes an asynchronous event stream $E=\{e_{i}\}$ with $e_{i}=(t_{i},x_{i},y_{i},p_{i})$, where $t_{i}$ is the timestamp, $\left(x_{i},y_{i}\right)$ the pixel location, and $p_{i}\in \{-1,+1\}$ the polarity. A single event does not track a pixel; object-level trajectories are constructed by associating time-ordered events across pixels according to the rules in Section 3.2.

To enhance the robustness of the tracking system, data augmentation is applied to the event stream [44], which includes translation and rotation transformations. The translation modifies the coordinates x and y by random values, expressed as $x^{\prime }=x+\Delta x$ and $y^{\prime }=y+\Delta y$, where Δx and Δy are random translation values. The rotation transformation is applied using a standard 2D rotation matrix to adjust the coordinates:

(1) $$\left[\begin{array}{c} x^{\prime }\\ y^{\prime } \end{array}\right]=\left[\begin{array}{cc} cos(\theta ) & -sin(\theta )\\ sin(\theta ) & cos(\theta ) \end{array}\right]\left[\begin{array}{c} x\\ y \end{array}\right]$$

where θ is a randomly selected rotation angle.

The object length is estimated by calculating the maximum Euclidean distance between any two points in the event trajectory:

(2) $$d_{\max}=\underset{i,j}{max}\left(\sqrt{{\left(x_{j}-x_{i}\right)}^{2}+{\left(y_{j}-y_{i}\right)}^{2}}\right)$$

where $\left(x_{i},y_{i}\right)$ and $\left(x_{j},y_{j}\right)$ are two distinct points in the event stream. For larger datasets, the bounding box diagonal is computed as:

(3) $$diagonal=\sqrt{{\left(x_{\max}-x_{\min}\right)}^{2}+{\left(y_{\max}-y_{\min}\right)}^{2}}$$

where $x_{\min},x_{\max},y_{\min},y_{\max}$ are the minimum and maximum coordinates of the object’s bounding box.

The quantities introduced earlier are used to scale and stabilise the kinematic estimates. Let the radius vector $r_{i}={\left[x_{i},y_{i}\right]}^{⊤}$ denote the spatial position of event $e_{i}$. The maximum trajectory length $d_{\max}$ in Equation (2) and the bounding-box diagonal in Equation (3) provide two scene-dependent scales. We normalise displacements and velocities by the diagonal so that all kinematic terms are measured per unit scene scale, and we cap neighbourhood searches and interpolation radii by a fraction of $d_{\max}$ to avoid drift on long tracks. Accordingly, Equations (4) and (5) are used in a discrete form consistent with the event scheme:

(4) $$v_{i}=\frac{r_{i}-r_{i-1}}{t_{i}-t_{i-1}}$$

(5) $$\;a_{i}=\frac{v_{i}-v_{i-1}}{t_{i}-t_{i-1}}$$

and their normalised counterparts are $\overset{\wedge }{v_{i}}=v_{i}/diagonal$ and $\overset{\wedge }{a_{i}}=a_{i}/\mathrm{diagonal}$. In practice we set the spatial search radius for supplementation to $\rho =\eta \min(d_{\max},\mathrm{diagonal})$ with a fixed η∈(0,1). With these definitions, Equations (4) and (5) describe the event trajectory as a time-ordered sequence $\left\{r_{i}\right\}$, while Equations (2) and (3) supply the reference scales that determine the magnitude and the admissible neighbourhood for interpolation and filtering. Interpolation declares a gap when the inter event interval exceeds three times the median per track, uses a spatial search radius of half the box diagonal, and a two-millisecond confirmation window, which limits corrections in size and prevents drift accumulation.

The Kalman filter is applied to predict the object’s future position using the following prediction step:

(6) $$x_{k|\;k-1}=Ax_{k-1|\;k-1}+Bu_{k}$$

Here $x_{k|\;k-1}$ denotes the a priori state estimate at time k given measurements up to k−1, that is, the prediction step. The subscript k is the time index and the bar k∣ k−1 indicates prediction from the previous step. The state $x_{k}$ stacks image-plane position and velocity as ${\left[x_{k},y_{k},v_{k}^{x},v_{k}^{y}\right]}^{⊤}$ and is propagated with a constant-velocity model with sampling interval Δt. The term $B_{k}u_{k}$ denotes a known exogenous control input such as commanded motion or IMU-derived acceleration expressed in the same kinematic units; when no control is available, $u_{k}=0$ and the prediction reduces to constant-velocity propagation with process noise.

Finally, the bounding box around the object is computed using:

(7) $$bounding\;box=(x_{min},y_{min},x_{max},y_{max})$$

where $x_{\min},x_{\max},y_{\min},y_{\max}$ are the minimum and maximum coordinates of the object’s bounding box. These bounding box coordinates are used to remap the events to the full-resolution image:

(8) $$x_{norm}=\frac{x-x_{min}}{x_{max}-x_{min}}\times (W-1),\;\;\;\;y_{norm}=\frac{y-y_{min}}{y_{max}-y_{min}}\times (H-1)$$

Here W denotes the image width and is unrelated to the trainable weights used later; learnable parameters are written in lowercase as w and H are the width and height of the full-resolution frame, and $x_{\mathrm{norm}}$ and $y_{\mathrm{norm}}$ are the normalised coordinates.

### 3.3. Training Framework for Event-Based Learning

The proposed training framework for event-based learning integrates several innovative modules that optimise the processing of event-driven data within a pure Spiking Neural Network (SNN) architecture. The primary innovation is the multi-scale event attention mechanism, which assigns importance to events based on their temporal and spatial relevance. For each event $e_{i}=(ts_{i},x_{i},y_{i},pol_{i})$, where $ts_{i}$ is the timestamp, $x_{i}$ and $y_{i}$ are the spatial coordinates, and $pol_{i}$ is the polarity, the temporal weight is calculated using the time difference between the event and its neighbours:

(9) $$Temporal\;Weight_{i}=exp\left(-\frac{\Delta t_{ij}}{\tau_{\mathrm{time}}}\right)$$

where $\Delta t_{ij}=|ts_{i}-ts_{j}|$ is the time difference between events $e_{i}$ and $e_{j}$, and $\tau_{\mathrm{time}}$ is a temporal scaling factor. The spatial weight is determined by the Euclidean distance between the spatial coordinates of the events:

(10) $$Spatial\;Weight_{i}=exp\left(-\frac{\Delta dist_{ij}}{\tau_{space}}\right)$$

where $\Delta dist_{ij}=\sqrt{{\left(x_{i}-x_{j}\right)}^{2}+{\left(y_{i}-y_{j}\right)}^{2}}$ is the spatial distance, and $\tau_{\mathrm{space}}$ is the spatial scaling factor. The parameters $\tau_{\mathrm{time}}$ and $\tau_{\mathrm{space}}$ set the temporal and spatial support of the attention and act as bandwidths for the respective kernels. Their values are selected on the training split by a small grid centred on sensor statistics; $\tau_{\mathrm{time}}\in \{3,5,7\}\mathrm{ms}$ and $\tau_{\mathrm{space}}\in \{1,2,3\}$ pixels, with $\tau_{\mathrm{time}}=5\;\mathrm{ms}$ and $\tau_{\mathrm{space}}=2$ pixels adopted unless stated otherwise. On the validation split, one factor sweeps around the selected scales showed stable optima. Performance varied by at most 0.3 percentage points for $\tau_{\mathrm{time}}$ and 0.4 percentage points for $\tau_{space}\;$ within the explored ranges. The polarity weight is based on whether the polarities of the two events match, expressed as:

(11) $$w_{\mathrm{pol}}(i,j)=\{\begin{array}{cc} 1, & p_{i}=p_{j},\\ \lambda , & p_{i}\neq p_{j}, \end{array}\;\;\;\;p_{i},p_{j}\in \{-1,+1\},0\leq \lambda <1.$$

Here $p_{i}$ and $p_{j}$ denote event polarities. The parameter λ is an attenuation factor for pairs of opposite polarity with 0≤λ<1 and is kept fixed within each experiment; the same setting is applied across all methods. This two-case definition makes the exclusivity explicit and removes the ambiguity that would arise from adding mutually incompatible conditions. The final attention weight is a weighted sum of these components:

(12) $$Attn\;Weight_{i}=a\times Temporal\;Weight_{i}+b\times Spatial\;Weight_{i}+g\times Polarity\;Weight_{i}$$

The coefficients α,β,γ weight the temporal, spatial and polarity terms in the composite attention. They are constrained to be non-negative and to sum to one and are chosen on the training split by a convex grid search; the equal setting $\alpha =\beta =\gamma =\frac{1}{3}$ is used when multiple settings perform similarly. Increasing α favours short, burst-like transients, increasing β favours coherent spatial structure, and increasing γ favours polarity consistency.

The attention weight determines the importance of each event in the network, guiding the model to focus on the most relevant events.

In addition, the spatio-temporal event convolution module is employed to extract key features from the event stream. This module applies convolution filters to the event data, allowing the network to capture spatial and temporal features such as edges and motion. The convolution operation is expressed as:

(13) $$Feature_{i}=\underset{k}{\sum }(E_{event}*K_{k})$$

where $E_{\mathrm{event}}$ represents the event stream, and $K_{k}$ are the convolution kernels. These kernels extract features from the event stream, which are then processed in the network.

Kernels adapt to DVS polarity and sparsity. ON and OFF branches share a 3 × 3 spatial kernel and a short causal temporal kernel. Each branch is softly gated by the local ON to OFF ratio, which amplifies the dominant polarity and suppresses the other without duplicating spatial parameters. Convolution is evaluated only on active voxels and their small neighbourhoods. Spatial dilation and temporal integration length are conditioned on local occupancy: sparse regions use larger dilation and longer integration, while dense regions use tighter dilation and shorter integration. Outputs from both branches are merged and normalised by the local event count to remove rate bias.

The Leaky Integrate-and-Fire (LIF) neuron model processes the events within the SNN. The membrane potential v(t) is updated according to the equation:

(14) $$\tau_{m}\frac{dV}{dt}=-\left(V-E_{L}\right)+R_{m}I_{\mathrm{syn}}(t)$$

Membrane potential V follows leaky integrate-and-fire dynamics with time constant $\tau_{m}=R_{m}C_{m}$ and leak reversal $E_{L}$. The synaptic drive $I_{\mathrm{syn}}(t)$ is a current that is converted to an equivalent voltage by the membrane resistance $R_{m}$, so all terms are expressed in volts.

Each event carries a composite attention weight according to Equation (12). During inference, the synaptic drive to a neuron is the weighted sum of its afferent event kernels, with each event contribution multiplied by its attention weight. Informative events deliver larger membrane increments and are more likely to reach the threshold. In contrast, events that are isolated in time or space, or that have inconsistent polarity, receive small weights and are effectively suppressed.

The training framework uses the Event-Weighted Spiking Loss (EW-SLoss) function to guide the weight update process. This custom loss function adjusts the weights based on the attention-weighted events, ensuring that the model focuses on the most relevant data during training. During learning the per event error is multiplied by the same attention weight so that gradients are concentrated on coherent structures and noisy updates are attenuated. The weight update rule is given by:

(15) $$\Delta w=-\eta \frac{\partial L_{\mathrm{EW}}}{\partial w}$$

Here w denotes any trainable parameter in the spiking recognition backbone and in the event-weighted readout; $L_{\mathrm{EW}}$ is the event-weighted spiking loss. Gradients are computed with a surrogate-gradient approximation to the spike nonlinearity, and parameters are updated as w←w+Δw.

## 4. Results

Inference was profiled with batch size one on a single CPU thread. The CPU was an Intel Core i9-13900K (Intel Corporation, Santa Clara, CA, USA) with a peak frequency of 5.8 GHz. Each slice contained approximately five to seven thousand events under the unified event-slicing protocol. DTEASN processes one sample in 28.0 ms on the CPU with an inference memory footprint of 46 MB. Average spike activity per sample is 5.4 × 10^3^. These measurements indicate that real-time inference is sustained on a single CPU core and that deployment does not rely on a discrete GPU.

### 4.1. Ablation Study

**Figure 3:**
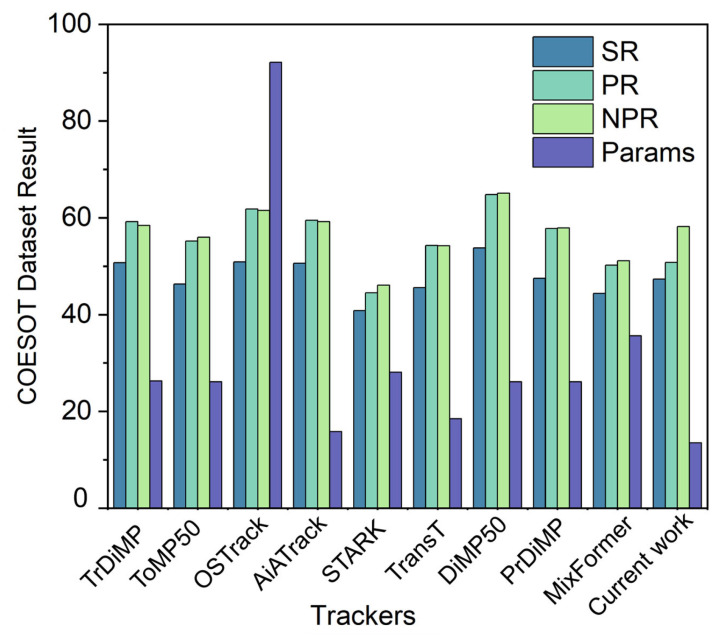
Event tracking performance on the COESOT dataset. The y axis denotes the result of SR/PR/NPR/Params. Resource related metrics are reported in Figure 4. The results correspond to the tracking branch only, evaluated with the official COESOT toolkit and the same initialization and search area for all methods.

**Figure 4:**
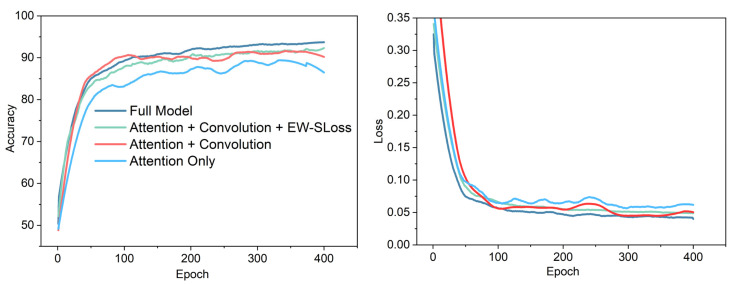
Training accuracy and loss curves of different configurations on the DVS Gesture dataset.

**Figure 5:**
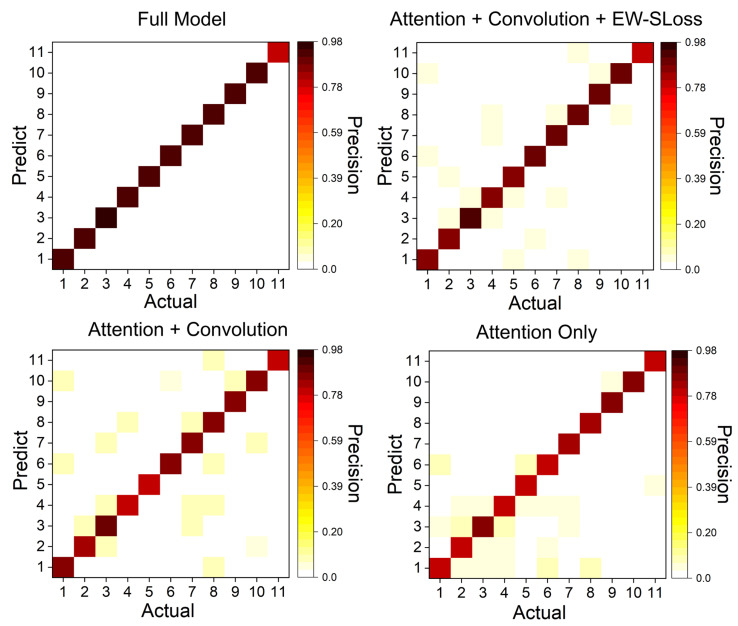
Confusion matrices for the four ablation models.

Ablation results on the DVS Gesture dataset [49] are reported in this section. The experiment methodically introduces diverse components of the model in order to evaluate their contribution to overall performance. The results are presented in three Figures. As illustrated in Figure 3, the performance of the event tracking system is presented. Figure 4 demonstrates the accuracy and loss curves, while Figure 5 presents the confusion matrix. The DVS-Gesture (DVS128) system comprises 11 distinct gesture classes, which have been recorded from 29 subjects under three distinct illumination conditions. The official subject split utilises 23 subjects for training and 6 subjects for testing, corresponding to 1176 training samples and 288 test samples in commonly used releases. In this study, validation is drawn from the training subjects for model selection, while results reported as “test” refer to the held-out test subjects/samples. Gesture classification results are reported on DVS-Gesture, whereas single-object tracking results are reported on COESOT; the two branches are evaluated independently in their respective settings.

Table 1 reports the validation sweep. The selected setting balances latency and feature completeness. Accuracy changed by at most 0.5 percentage points around the selected setting. Longer windows increased decision delay without accuracy gain, while shorter windows were faster but lost fine transients.

All numbers are averaged over three runs on the CPU with batch size one and fixed seeds. Forward time measures only the model pass without data loading. In the variant without the tracking mechanism, associations are produced by a greedy IoU matcher between consecutive slices with an IoU threshold of 0.5 and a one-to-one constraint. No motion gating or re identification is used and a track stops when no match is found.

To isolate the remaining core innovations, Table 2 ablates the lightweight event tracking mechanism and the custom synaptic connection rule. Computation time, tracking success rate, spikes per inference, and accuracy per million spikes are reported on the same validation split. Removing the tracking mechanism reduced computation slightly but caused a clear drop in tracking success. On digital neuromorphic chips, power scales with spike activity and recent measurements place per spike energy in the low tens of picojoules. Using 22 to 24 picojoules per spike, the estimated energy per inference is about 28 microjoules at 1.20 million spikes, 29 microjoules at 1.22 million, 44 microjoules at 1.85 million, and 45 microjoules at 1.90 million. Removing the synaptic rule increased spikes and reduced spike efficiency without accuracy gain.

Robustness to missing events is reported in Table A1 in the Appendix A. Performance remained stable up to ten percent and degraded only slightly at twenty percent.

A sensitivity analysis varied $\tau_{\mathrm{time}}$ and $\tau_{\mathrm{space}}$ while all other settings were fixed. Validation accuracy changed by at most 0.3 percentage points across $\tau_{\mathrm{time}}$ and 0.4 percentage points across $\tau_{\mathrm{space}}$ near the selected values and degraded monotonically only beyond the grid bounds.

**Figure 6:**
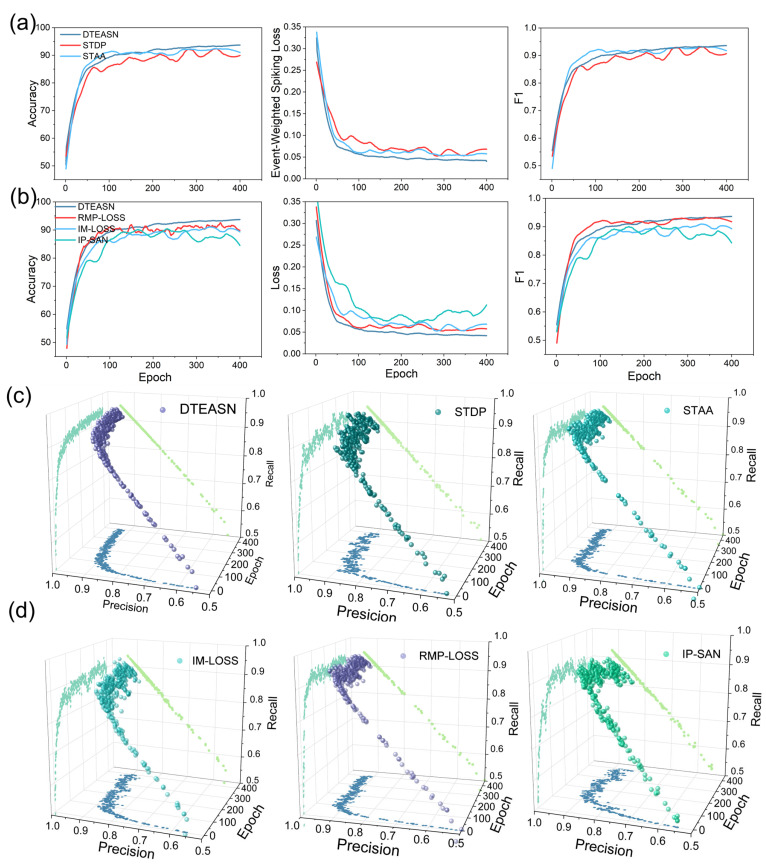
Training dynamics and 3D precision–recall trajectories. (**a**) Accuracy, event-weighted spiking loss, and F1 versus epoch for DTEASN, STDP, and STAA. (**b**) The same metrics for DTEASN, RMP-LOSS, IM-LOSS, and IP-SAN. (**c**,**d**) For each method, a 3D trajectory in the Precision–Recall–Epoch space is shown (solid markers), together with its orthogonal projections onto the three coordinate planes: Precision–Recall (light-green trace on the top plane), Precision–Epoch (blue trace on the bottom plane), and Recall–Epoch (faint diagonal trace). The projections use distinct colours to distinguish them from the main 3D path, and all axes share identical ranges across subplots for direct comparison.

Figure 3 reports single-object tracking results on COESOT [22] using the standard SR, PR and NPR metrics [50]. The tracking branch is evaluated independently under the official COESOT evaluation settings with identical initialization and search area across all trackers. Despite the fact that the Our Tracker does not achieve the highest SR/PR/NPR (with AiATrack ranking first on all three metrics), it has the smallest parameter count (Params) of the methods compared, including TrDiMP [15], ToMP50 [51], OSTrack [52], AiATrack [15], STARK [53], TransT [5], DiMP50 [54], PrDiMP [10], MixFormer [10], and Our Tracker. This compact model footprint substantially reduces memory and computational demand, offering a favourable accuracy-efficiency trade-off for real-time, resource-constrained deployment. In practice, although the absolute accuracy lags the best performer by a modest margin, the pronounced efficiency advantage renders Our tracker highly practical for event-driven tracking scenarios. Recent frameworks on COESOT are included as reference points in Figure 3. Aitsam [39] focuses on neuromorphic multi-object tracking with hardware-centred metrics that differ from those of the single-object COESOT protocol, which reports success rate, precision and normalised precision. For this reason, they are cited for context rather than being tabulated in Figure 3. Subset results for low light, cluttered background, and two object cases are summarised in Table A2 in the Appendix A under the same protocol. Resource requirements and training curves are presented separately in Figure 4 (and Figure 6 where applicable) and are not derived from Figure 3.

Qualitative tracking observations on COESOT are summarised here. Successful cases include low-light scenes and rapid changes in scale and aspect, where polarity-consistent clusters support stable updates. Typical failures involve sustained occlusion or near-duplicate distractors, where short drift or identity switches may occur when clusters merge. These observations are consistent with the subset deltas reported in Table A2.

The training accuracy and loss curves for the various configurations on the DVS Gesture dataset are presented in Figure 4. The Full Model (DTEASN) attains the maximum accuracy of 95.16%, thereby demonstrating a substantial improvement over alternative configurations. Attention + Convolution + EW-SLoss demonstrated an accuracy of 93.27%, while Attention + Convolution and Attention Only exhibited lower accuracy of 91.81% and 87.63%, respectively. In terms of loss, the Full Model demonstrates the lowest loss, indicating efficient learning and convergence. Notably alternative configurations, such as Attention + Convolution + EW-SLoss, show enhanced performance. Conversely, the Attention Only model exhibits the highest loss, indicating that the absence of spatio-temporal event convolution and EW-SLoss impedes effective learning. The findings underscore the efficacy of the Full Model, underscoring that incorporating spatio-temporal convolution and Event-Weighted Spiking Loss (EW-SLoss) leads to substantial enhancements in both accuracy and loss reduction, thereby validating the approach’s superiority.

As illustrated in Figure 5, the confusion matrices provide a comparative analysis of the four ablation variants, with the variants presented from left to right. Full Model, Attention + Convolution + EW-SLoss, Attention + Convolution, Attention Only). The Full Model displays the most pronounced diagonal, with per-class precision ranging from 0.90 to 0.98 and off-diagonal entries generally not exceeding 0.03, suggesting well-separated classes. The incorporation of EW-SLoss effectively suppresses spurious activations relative to Attention + Convolution (off-diagonals predominantly 0.03–0.05). The elimination of EW-SLoss and the synaptic mechanism results in an augmentation of cross-class interference, particularly among semantically related categories. The Attention Only variant exhibits the most dispersed errors, with several off-diagonal elements reaching 0.07–0.08 and diagonal values dropping to approximately 0.80–0.85. The left-to-right progression demonstrates consistent gains from each component, with the Full Model exhibiting the most uniform per-class precision and the lowest misclassification.

Representative qualitative classification observations are summarised here. Correct predictions typically exhibit compact, polarity-consistent clusters aligned with the gesture trajectory, whereas residual errors arise between gestures that share similar motion primitives or partially overlapping temporal phases. The attention mechanism and the event-weighted loss suppress isolated spikes and reduce spurious activations, which matches the off-diagonal patterns in Figure 5.

### 4.2. Comparison of DTEASN with Conventional Spiking Algorithms

This section will compare the performance of DTEASN with conventional spiking neural network (SNN) models, including STDP [8], STAA [55], RMP-LOSS [56], IM-LOSS [56] and IP-SAN [57], across multiple performance metrics. As illustrated in Figure 6a, a comparison is made between the training accuracy, loss, and F1 score for the various models. While the STAA model demonstrates the most rapid enhancement in accuracy during the training phase, the DTEASN model attains the optimal accuracy, minimal loss, and maximum F1 score by the conclusion of the training process. This finding suggests that the incorporation of the spatio-temporal synaptic mechanism within DTEASN leads to a substantial enhancement in learning efficiency and overall performance when compared with STDP and STAA. Figure 6b provides a comparison of DTEASN’s Event-Weighted Spiking Loss (EW-SLoss) with other loss functions, including RMP-Loss, IM-Loss, and IP-SAN. The findings indicate that EW-SLoss results in the minimisation of training loss, while concurrently achieving optimal performance metrics, including accuracy and the F1 score. This outcome serves to substantiate the efficacy of EW-SLoss in enhancing convergence speed and model learning dynamics. This emphasises the pivotal function of EW-SLoss in optimising the efficacy of the model in comparison to alternative loss functions.

As illustrated in Figure 6c,d, the 3D plots depict precision, epoch, and recall metrics for DTEASN in comparison to alternative models. As illustrated in Figure 6c, the x-y plane (precision vs. recall) demonstrates that DTEASN consistently exhibits higher precision and recall across epochs in comparison to STDP and STAA. The data points for DTEASN are predominantly concentrated in the upper-right quadrant, suggesting superior precision and recall. In the x–z plane (precision vs. epoch), DTEASN demonstrates a steady increase in precision, while STDP and STAA exhibit more erratic performance. In a similar manner, the y–z plane (recall vs. epoch) demonstrates DTEASN’s consistent enhancement in recall, whilst STDP and STAA exhibit variable recall. As demonstrated in Figure 6d, the 3D visualisations for the loss function comparison substantiate that DTEASN with EW-SLoss sustains a discernible upward trend in precision, recall, and epoch, eclipsing other loss functions such as RMP-Loss, IM-Loss, and IP-SAN.

**Figure 7:**
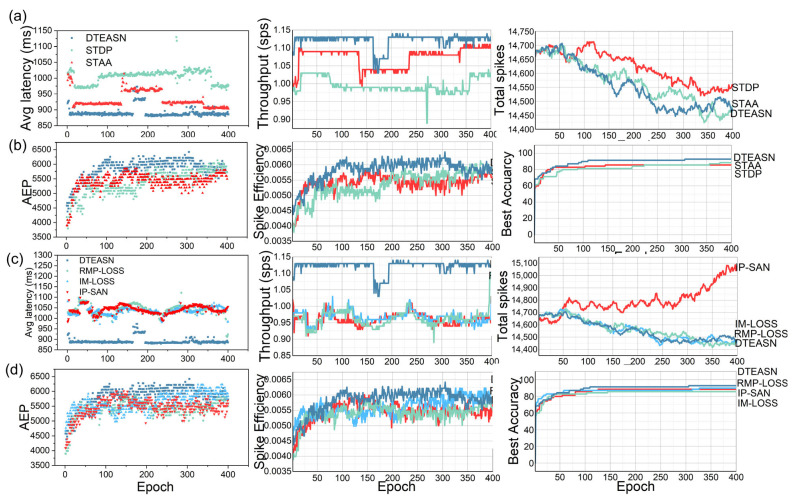
Performance Comparison of DTEASN with Conventional Spiking Algorithms and Loss Functions. (**a**) Comparison of average latency, throughput (sps), and total spikes for different synaptic algorithms (DTEASN, STDP, STAA). (**b**) Comparison of Average Event Processing (AEP), spike efficiency, and best accuracy for different synaptic algorithms. (**c**) A 3D visualisation of precision, epoch, and recall for different synaptic algorithms (DTEASN, STDP, STAA). (**d**) A 3D visualisation of precision, epoch, and recall for different loss functions (EW-SLoss, RMPLoss, IMLoss, IP-SAN).

As illustrated in Figure 7, the proposed model exhibits several key efficiency attributes, including low latency, compact spiking activity, and a pure SNN pipeline devoid of tensor/frame-based operations. As illustrated in Figure 7a (synaptic algorithms), a comparative analysis is conducted among DTEASN, STAA, and STDP, with metrics including average latency, throughput (spikes per second, SPS), and total spikes. It is evident that DTEASN attains the minimum latency, which is approximately 5% lower than STAA and 11.11% lower than STDP. Concurrently, it delivers the highest SPS across all epochs. It is noteworthy that, despite the augmented throughput, the aggregate spike count of DTEASN persists at a low and proximate level to STAA (marginally lower), thereby demonstrating effective event utilisation. Figure 6b further examines Average Event Processing (AEP), spike efficiency, and best accuracy under the same synaptic setups; DTEASN leads on all three curves, indicating superior event handling efficiency and the highest peak accuracy among the compared synaptic algorithms.

The same set of metrics is reassessed for loss functions in Figure 7c,d, contrasting our EW-SLoss with IM-LOSS, RMP-LOSS, and IP-SAN. As demonstrated in Figure 7c, DTEASN with EW-SLoss exhibits the lowest average latency, the highest throughput, and lower total spikes in comparison to the alternative losses, thereby reinforcing its favourable accuracy-efficiency trade-off. As illustrated in Figure 7d, the AEP, spike efficiency, and best-accuracy trajectories once again indicate a preference for EW-SLoss, which achieves the highest spike efficiency and the most optimal best-accuracy curve while preserving stable growth across epochs. The collective analysis of these panels substantiates that DTEASN achieves low-latency, resource-frugal event processing as a pure SNN, outperforming both conventional synaptic rules and competing loss formulations on efficiency-critical indicators.

### 4.3. Classification Accuracy on Datasets

As illustrated in Table 3, the classification accuracy of representative models across datasets is presented, and a comparison is made between DTEASN and recent methods. On the DVS-Gesture dataset, DTEASN achieves a 95.16% success rate, surpassing KLIF at 94.10% and CSNN at 93.40%. On the ES-ImageNet dataset, DTEASN achieves a 44.69% success rate, surpassing Bridge Conversion (43.74%) and ConvECLIF2D-A (44.25%). On the CIFAR-10-DVS dataset, DTEASN attained an accuracy of 75.20%, surpassing the PLIF at 74.80% and the SEW ResNet at 74.40%. On the MNIST dataset, DTEASN achieved a 98.13% success rate, which is lower than the 98.80% achieved by DTIF and slightly below the 98.89% achieved by STBP, while remaining above the 97.43% success rate of EP-SNN. The lower value on MNIST is attributable to the dataset’s minimal temporal structure, which favours longer temporal integration and stronger spatial inductive bias. DTIF employs a LeNet-style backbone with 90 time steps that benefits static digits, whereas DTEASN operates with 20 time steps and prioritises low latency and sparse spiking. The event-aligned attention and the event-weighted loss provide limited additional signal on static images, producing a modest accuracy–efficiency trade-off consistent with the design goals.

Comparability note: Table 3 summarises representative SNN models to contextualise design choices. Because several entries target different objectives from dynamic recognition and tracking, the table is descriptive rather than a head-to-head benchmark. Objective, dataset and protocol differences are stated explicitly, and numbers quoted from original papers are marked accordingly.

## 5. Conclusions

This paper presented the Dynamic Tracking with Event Attention Spiking Network (DTEASN), a pure spiking framework for efficient, real-time event-based tracking and recognition. By removing conventional CNN operations and aligning computation with the asynchronous characteristics of Dynamic Vision Sensors, the approach reduces GPU dependency while preserving low latency. The design combines an event-driven multi-scale attention module with a spatio-temporal event convolver to enhance feature extraction under sparse, polarity-coded streams, and the Event-Weighted Spiking Loss (EW-SLoss) prioritises salient spatio-temporal events during learning. A lightweight tracking mechanism further sustains real-time throughput with minimal computational overhead. The experimental results indicate favourable accuracy with reduced operation counts and improved energy efficiency relative to competing methods, consistent with sensor-centric deployment requirements. Future work will focus on scaling to more complex datasets and longer sequences, and on integrating the pipeline with neuromorphic or low-power edge hardware to obtain measured on-chip latency and energy for further optimisation.

## Figures and Tables

**Table 1:** Sensitivity of window and step on the validation split.

Window Length, ms	Step 5 ms	Step 10 ms	Step 15 ms
10	94.6	94.8	94.7
20	95.0	95.1	94.9
30	94.9	95.0	94.7
Decision delay, ms	38	48	58
Update rate, Hz	200	100	67

Notes. Decision delay equals the window length plus the measured computation time of 28 ms. Update rate equals one over the step size.

**Table 2:** Ablation of the event tracking mechanism and the custom synaptic rule on the validation split. Lower time and spikes are better.

Variant	Computation Time ms	Tracking Success Rate %	Spikes Per Inference M	Accuracy Per M Spikes
Full model	28.0	95.4	1.20	79.5
Without tracking mechanism	26.5	89.1	1.22	73.0
Without synaptic rule	29.2	92.3	1.85	49.9
Without both	27.0	86.7	1.90	45.6

Notes. Tracking success rate counts tracks that remain correct for at least ten frames with intersection over union greater than 0.5 on the validation split. Spikes per inference is the total emitted spikes across all spiking layers for one forward pass. Accuracy per million spikes is accuracy divided by spikes in millions. The same matcher is used to compute the tracking success rate for all variants. For the no tracking variant this is the greedy IoU baseline described above.

**Table 3:** Test performance of different models on the conventional datasets (MNIST, CIFAR-10-DVS, ES-ImageNet, and DVS-Gesture datasets, respectively).

Image Dataset	Method	Spiking Network	Neuron Model	Time Steps	Accuracy (%)
DVS-Gesture	KLIF [58]	Modified PLIF Net	KLIF	12	94.10
	Rate-LOSS RNN [59]	Input-MP4-64C3-128C3-AP2-128C3-AP2-256FC-11	RNN	5	90.27
	R-SAN [60]	Input-MP4-64C3-128C3-AP2-128C3-AP2-256FC-11	LIF	5	91.77
	CSNN [61]	Input-MP4-64C3-128C3-AP2-128C3-AP2-256FC-11	LIF	5	93.40
	Slayer [62]	8-layers convolution	-	5	93.64
	LIF [63]	5-layer convolution	LIF	16	93.75
	This Work	DTEASN	LIF	12	95.16
ES-ImageNet	Bridge Conversion [64]	ResNet-34	-	8	43.74
	ConvECLIF2D-A [65]	ResNet-18	-	8	44.25
	ES-ImageNet [66]	ResNet-18	-	8	43.42
	This Work	DTEASN	LIF	8	44.69
CIFAR-10-DVS	PLIF [67]	PLIF Net	PLIF	20	74.80
	TDBN [68]	ResNet-19	-	10	67.80
	KLIF [58]	Modified PLIF Net	KLIF	15	70.90
	SEW ResNet [68]	Wide 7B Net	LIF	16	74.40
	This Work	DTEASN	LIF	20	75.20
MNIST	Natural ANN [69]	Input-800FC-10	-	-	98.52
	EP SNN [70]	Input-MP4-64C3-128C3-AP2-128C3-11	LIF	20	97.43
	DTIF [71]	Lenet5	DTIF	90	98.8
	STBP [72]	Input-800FC-10	BIF	35	98.89
	This Work	DTEASN	LIF	20	98.13

Table note. Accuracy (%) is defined as top-1 classification accuracy on the validation split, computed as the number of correctly classified instances divided by the total number of instances.

## Data Availability

The code and trained models are available at: https://github.com/2221d/DTEASN (accessed on 1 August 2025).

## Appendix A

### Appendix A.1

**Table A1:** Robustness to missing events on the validation split. Lower drift is better.

Missing Events %	Mean Drift px	Tracking Success Change pp
0	0.0	0.0
5	0.1	−0.3
10	0.3	−0.6
20	0.7	−1.2

Notes. Events were randomly dropped at five, ten, and twenty percent on the validation split while all other settings were fixed and interpolation was enabled. Drift is the absolute deviation of the track centre over gaps. Tracking success uses the same definition as in Section 4.1. Forward time changed by at most 0.1 milliseconds across these settings.

**Table A2:** COESOT subsets under the same protocol. Deltas relative to the full set are reported in percentage points.

Subset	ΔSR	ΔPR	ΔNPR
Low light	−0.8	−1.0	−1.1
Cluttered	−0.7	−1.2	−1.5
Two object	−1.6	−1.9	−1.8

Notes. Events were randomly dropped at five, ten, and twenty percent on the validation split while all other settings were fixed and interpolation was enabled. Drift is the absolute deviation of the track centre over gaps. Tracking success uses the same definition as in Section 4.1. Forward time changed by at most 0.1 milliseconds across these settings.

**Table A3:** Reproducibility details used in all experiments.

Item	Setting
Optimiser	AdamW
Base learning rate	0.001
Learning rate schedule	Cosine decay to 0.000001 with five epoch warm up
Epochs	120
Batch size	16
Weight decay	0.0001
Gradient clipping	Global norm 1.0
Seed	42
Checkpointing	Best by validation accuracy with periodic saves
Latency protocol	Batch size one, forward only, warm up discarded, averages over repeated iterations, device synchronisation before and after model call for GPU
Hardware reference	Same machine as in the Experiment section
Event slicing	Window 20 milliseconds, step 10 milliseconds
Polarity handling	Two separate channels for ON and OFF
Timestamp normalisation	Referenced to the start of each window and divided by the window length
Voxelisation	Causal grid over space and time with polarity channels
Isolated event removal	Remove single events without a neighbour within one pixel and two milliseconds
Count clipping	Per location event counts clipped to a maximum of 8
Input normalisation	Divide by the local event count

**Table A4:** Summary metrics for the four ablation models on the validation split.

Model	Precision	Recall	F1	Max Test Accuracy	Min Test Loss
Attention Only	0.82	0.81	0.81	86.33	0.00218
Attention + Convolution	0.85	0.83	0.84	89.75	0.00174
Attention + Convolution + EW-SLoss	0.89	0.87	0.88	91.20	0.00137
Full Model	0.95	0.93	0.94	94.31	0.00098

Notes. Precision = TP/(TP + FP); Recall = TP/(TP + FN); F1 = 2·Precision·Recall/(Precision + Recall); Accuracy = total correct/total samples.

### Appendix A.2

Appendix A.2 reports additional quantitative results on COESOT. Table A5 lists public results under the official SR, PR, and NPR metrics to complement the analysis in the main text.

**Table A5:** Event tracking performance on the COESOT dataset.

Method (Venue, Year)	Modality	SR	PR	NPR	Params
DTEASN, tracking branch	Event-only	41	57	61	7
HDETrack (CVPR 2024)	Event-only	53.1	64.1	64.5	≈92
CEUTrack-L + Orthogonal High-Rank Aug. (ICCV 2023)	RGB + Event (EVox)	65.0	73.8	71.9	(not reported; depends on base backbone)

Notes. Metrics follow the COESOT evaluation (SR/PR/NPR) used in Figure 3; modality and settings are unchanged from the main figure.
